# Immune Trait Shifts in Association With Tobacco Smoking: A Study in Healthy Women

**DOI:** 10.3389/fimmu.2021.637974

**Published:** 2021-03-09

**Authors:** Giulia Piaggeschi, Simona Rolla, Niccolò Rossi, Davide Brusa, Alessio Naccarati, Simon Couvreur, Tim D. Spector, Mario Roederer, Massimo Mangino, Francesca Cordero, Mario Falchi, Alessia Visconti

**Affiliations:** ^1^Italian Institute for Genomic Medicine, c/o IRCCS Candiolo, Turin, Italy; ^2^Department of Computer Science, University of Turin, Turin, Italy; ^3^Department of Clinical and Biological Sciences, University of Turin, Turin, Italy; ^4^Department of Twin Research and Genetic Epidemiology, King's College London, London, United Kingdom; ^5^Institute of Experimental and Clinical Research, Université Catholique de Louvain, Brussels, Belgium; ^6^Candiolo Cancer Institute, Fondazione del Piemonte per l'Oncologia-Istituto di Ricovero e Cura a Carattere Scientifico (FPO-IRCCS), Turin, Italy; ^7^Vaccine Research Center, National Institutes of Health, Bethesda, MD, United States; ^8^National Institute for Health Research (NIHR) Biomedical Research Centre at Guy's and St Thomas' Foundation Trust, London, United Kingdom

**Keywords:** tobacco smoking exposure, immune traits, leukocyte-shift, immune cell subset frequencies, epidemiology

## Abstract

Tobacco smoking is known to impact circulating levels of major immune cells populations, but its effect on specific immune cell subsets remains poorly understood. Here, using high-resolution data from 223 healthy women (25 current and 198 never smokers), we investigated the association between smoking status and 35,651 immune traits capturing immune cell subset frequencies. Our results confirmed that active tobacco smoking is associated with increased frequencies of circulating CD8+ T cells expressing the CD25 activation marker. Moreover, we identified novel associations between smoking status and relative abundances of CD8+ CD25+ memory T cells, CD8+ memory T cells expressing the CCR4 chemokine receptor, and CD4+CD8+ (double-positive) CD25+ T cells. We also observed, in current smokers, a decrease in the relative frequencies of CD4+ T cells expressing the CD38 activation marker and an increase in class-switched memory B cell isotypes IgA, IgG, and IgE. Finally, using data from 135 former female smokers, we showed that the relative frequencies of immune traits associated with active smoking are usually completely restored after smoking cessation, with the exception of subsets of CD8+ and CD8+ memory T cells, which persist partially altered. Our results are consistent with previous findings and provide further evidence on how tobacco smoking shapes leukocyte cell subsets proportion toward chronic inflammation.

## Introduction

According to the 2019 World Health Organization report, tobacco smoking kills more than eight million people every year (1), and its association with the development of several pathologies, such as respiratory, cardiovascular, neurological, and autoimmune diseases, as well as of several types of cancer, is well established (2–4).

In healthy individuals, tobacco smoking alters leukocyte cells count and distribution in peripheral blood, with several studies showing, in both sexes, an increase in the total number of leukocytes in current smokers compared to former and never smokers (5–7). The mechanisms underlying this alteration are not fully understood yet, and studies investigating the effect of smoking on the immune system led to conflicting conclusions (8, 9). The leading hypothesis suggests that the irritating effect of tobacco smoke on the respiratory tract results in the release of pro-inflammatory cytokines, such as TNF-α, IL-1, IL-6, IL-8, and granulocyte-macrophage colony-stimulating factor, which can, in turn, increase the number of leukocytes (7). Alternatively, it has been suggested that nicotine-induced release of hormones from the adrenal gland (catecholamines) can stimulate cortisol secretion and thus inhibit antibody responses, T cell proliferation, and neutrophilic phagocytic activity (5, 6).

So far, studies have investigated the effect of tobacco smoking only on primary leukocyte subsets (e.g., T cells, B cells, natural killer cells, monocytes, and granulocytes), showing a decrease in circulating natural killer cells (10), an increase of CD3+ (11), CD4+ (helper) (5, 12), memory and naïve T cells (13) in current compared to never smokers. A decrease of T-helper cells and an increase of CD8+ (cytotoxic) T cells, corresponding to a decreased CD4+/CD8+ ratio, in current compared to never smokers has also been reported (14), albeit this observation was then contradicted in another study (15). Yet, a finely detailed investigation of the effect of smoking on more specific leukocyte subsets is still lacking.

**Figure 1:**
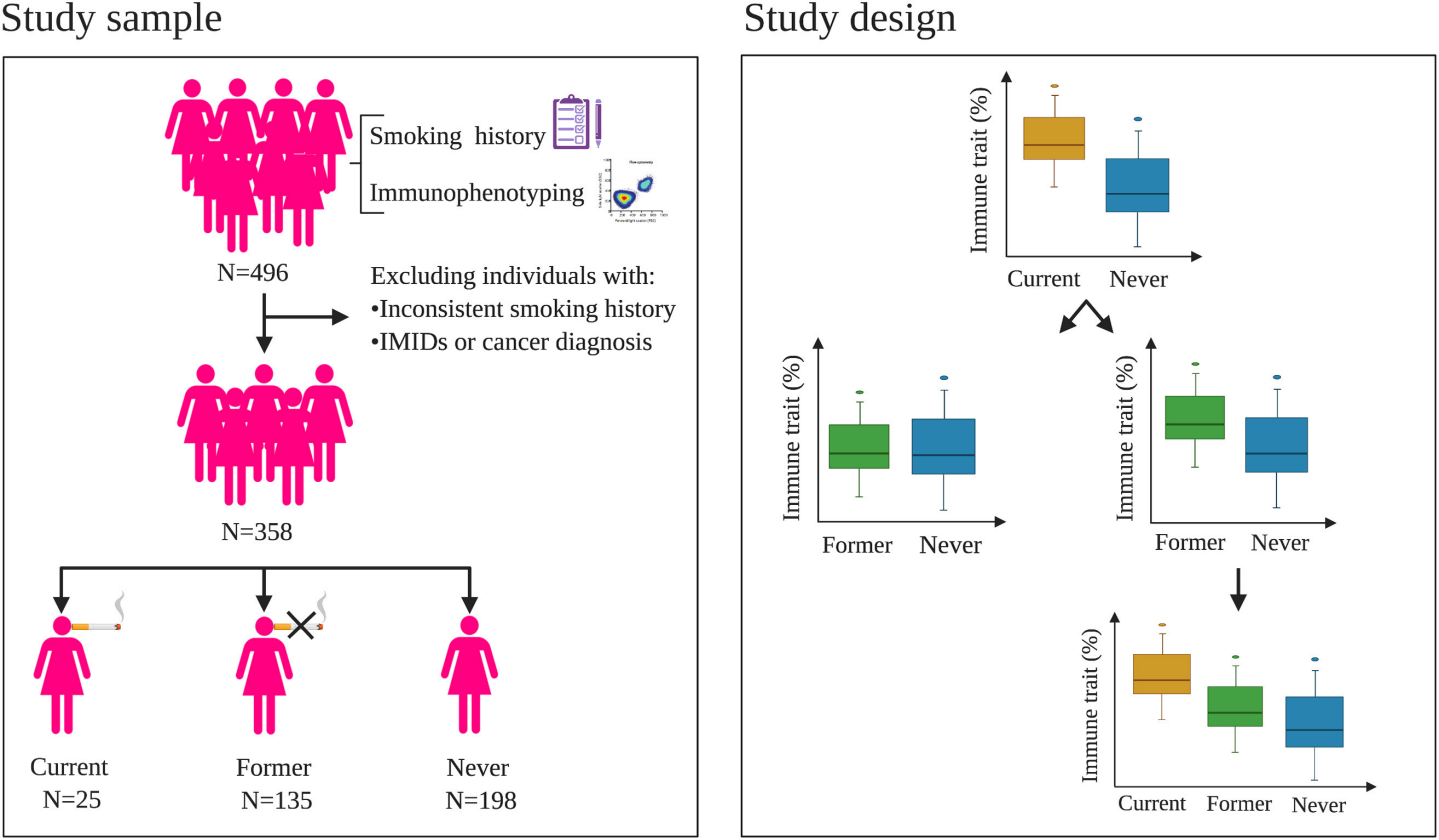
Study sample and design. **Left panel**: criteria used to select the women included in the study. Starting from 496 women with immunophenotyping and smoking history, 358 subjects were selected to be part of the study, which included 25 current, 135 former, and 198 never smokers. **Right panel**: analysis design. First, we aimed at identifying differences in the immune trait relative proportions between current and never smokers. Then, using data from former smokers, we investigated whether the relative proportion of these significantly different immune traits were fully or partially restored to never-smoker frequencies after smoking cessation. IMIDs: immune-mediated inflammatory diseases.

In this study, using information from 358 females from the TwinsUK cohort, we investigated: (a) the association between active tobacco smoking and 35,651 immune traits capturing immune cell subset frequencies (CSFs) and (b) whether the relative frequencies of these associated immune traits show evidence of restoration to non-smoking frequencies after smoking cessation. Study sample and design are summarized in Figure 1.

## Materials and Methods

### Study Population

The TwinsUK cohort includes about 14,000 subjects, with a large prevalence of women, showing disease and lifestyle characteristics similar to the general UK population (16, 17). St. Thomas' Hospital Research Ethics Committee approved the study, and all twins provided written informed consent.

### Immunophenotyping

A full description of the quantification of the immune cells and their phenotype is detailed elsewhere (18, 19). Briefly, leukocytes were characterized in 497 females from the TwinsUK cohort using cryopreserved PBMC samples. All samples were analyzed by flow cytometry on seven distinct 14-color panels (summarized in Supplementary Table 1) with an optimized staining procedure (18). Molecular markers were purchased from BectonDickinson, BioLegend, eBiosciences, Life Technologies, Beckman Coulter, Fisher Scientific, or Miltenyi Biotec; or conjugated in house. A full description of gating strategy for each panel is reported in (18), except for panel 6, reported in (19). In the first stage, parent lineages were defined within each panel via manual gating based on canonical marker combinations for leukocyte subsets of known functionality. Within each parent lineage, boolean gates were then manually defined for each additional cell surface marker (i.e., markers not used to determine the parent lineage), and information on all combinations of these boolean gates (i.e., whether positive, negative or ignored) were subsequently used to define subsets of immune cells and to measure their immune cell subset frequencies (CSFs, evaluated as percentages with respect to the total number of leukocytes in their parent lineage). Using this approach, we captured a grand total of 88,367 immune traits, describing 50 supersets of the parent lineages, and 88,317 CSFs.

To select 35,651, robust immune traits, we applied several criteria. First, we removed 41,336 CSFs with median value <0.1 or >99%, as done previously (18, 19). Second, we removed 34 redundant CSFs which passed the previous quality check steps and were measured in multiple panels (median Spearman's ρ within pairs = 0.88, range = 0.37–0.95). Third, we removed 11,296 CSFs strongly deviating from normality.

Immune traits were log-transformed, to improve the normality of their distribution, and then corrected for batch effects using a linear mixed model, as implemented in the R package *lme4* (v1.1.21) with flow cytometry batch number included as a random effect. Before carrying out the association analyses, we removed outliers (i.e., immune trait measurements deviating more than three standard deviations from the mean of each trait).

### Self-Reported Smoking History

Detailed information about smoking history was self-reported via 11 longitudinal questionnaires, collected from 1992 to 2010 in 496 individuals with immunophenotyping available (median number of responses: 7). Consistency of self-reported smoking status was assessed using additional self-reported information, i.e., age of start and quitting smoking, and the number of cigarettes and/or packs smoked. For instance, individuals who described themselves as never smokers, but reported, in any questionnaire, age of start and/or quitting smoking, and/or that they had smoked any number of cigarettes were removed from this study. We allowed for smoking relapse after smoking cessation and considered as current status the latest reported before immunophenotyping. This resulted in the inclusion of 460 individuals, 35 of whom were current smokers, 189 former smokers, and 236 reported never having smoked.

History of immune-mediated inflammatory diseases (IMID, i.e., chronic obstructive pulmonary disease, Crohn's disease, systemic lupus erythematosus, multiples sclerosis, polymyalgia rheumatica, psoriatic arthritis, rheumatoid arthritis, and ulcerative colitis) was traced through 15 longitudinal self-administered questionnaires completed between 2004 and 2017 (median number of responses per individual: 3). For each condition, study subjects who reported being diagnosed by a doctor at least once were treated as IMID cases, and when multiple ages at first diagnosis were provided, the minimum age was considered. Cancer history was available from the 2019 Office for National Statistics. Non-melanoma skin cancers and carcinomas *in situ* were not taken into account. Using these pieces of information, 102 individuals were excluded either because having a diagnosis of IMID reported before or within 2 years from immunophenotyping or being diagnosed for one or more cancers dating 5 years before or within 1 year from immunophenotyping.

The final dataset consisted of 358 healthy female individuals, 25 of whom were current smokers, 135 former smokers, and 198 never smokers (Figure 1, left panel; Supplementary Table 2).

### Lifestyle Factors

Height and weight were measured for all individuals included in this study during twins' clinical visits at King's College London. Individuals were asked to remove their shoes, and height (in cm) was measured using a stadiometer, while weight (in kg) was measured on digital scales.

Socioeconomic status was measured using the Index of Multiple Deprivation (IMD) based on the postcode (or UK grid reference mapped to postcode) where an individual lived at the time or near the time of sample collection (20), which was available for 344 individuals included in this study (24 current, 126 former, and 194 never smokers). IMD values range from 1 (=more deprived) to 5 (=less deprived).

Alcohol consumption was calculated using UK food composition table from 131-item self-administered food-frequency questionnaires established for the EPIC-Norfolk study (21), which were collected within 5 years from immunophenotyping. It was available for 320 individuals included in this study (20 current, 123 former, and 177 never smokers).

### Statistical Analyses

First, we aimed at identifying the immune traits involved in the response to active smoking using data from current and never smokers (Figure 1, right panel). Due to the high variability of time of smoking cessation before immunophenotyping (range: 1–50 years), we excluded former smokers from this analysis to avoid any confounding effects. Associations of immune traits with smoking status were carried out using a linear mixed model, as implemented in the *lmerTest* R package (function *lmer*, v3.1.1), including age at immunophenotyping as a fixed effect, and family as a random effect, to correct for the non-independence of the twin observations.

Due to the strong correlation among immune traits, we considered as significant the associations passing a Bonferroni-derived threshold of 0.05/*N*_eff_, where *N*_eff_ is the effective number of independent tests calculated on the whole set of 497 individuals with immunophenotyping data using the approach proposed by Li and Ji (22).

Due to the unequal sample size between current and never smokers, for each of the *N* immune traits passing the Bonferroni-derived threshold of 0.05/*N*_eff_ described above, we generated 5,000 random datasets where labels indicating smoking status were randomly permuted between monozygotic/dizygotic twin pairs and among singletons, in order to preserve the family structure and, thus, the underlying genetic correlation. Then, we counted the number of times *T* the association *p*-value in the random datasets was lower than 0.05/*N*_eff_, and used this number to evaluate an empirical *p*-value as (*T* + 1)/5,001. We confirmed an association as significant when its empirical *p*-value passed a Bonferroni-derived threshold of 0.05/*M*_eff_, where *M*_eff_ is the effective number of independent tests evaluated by the approach proposed by Li and Ji on the subset of the *N* significantly associated immune traits.

Then, to rule out the presence of a confounding effect due to alcohol consumption, we investigated whether alcohol consumption was associated with the identified immune traits using the statistical model described above, and considered as significant associations passing a Bonferroni-derived threshold of 0.05/*M*_eff_.

Next, we investigated whether the immune traits associated with active smoking remained altered after smoking cessation using data from former and never smokers and the statistical model described above (Figure 1, right panel**)**. Finally, to investigate the presence of a trend in the relative frequencies of the altered immune traits in current vs. former vs. never smokers, we performed a further association study including the three smoking categories (i.e., current, former, and never smokers; Figure 1, right panel), following the statistical model detailed above. In both analyses, associations passing a Bonferroni-derived threshold of 0.05/*M*_eff_ were considered as significant.

## Results

This study included 358 healthy women of European ancestry (43 and 94 monozygotic and dizygotic twin pairs, respectively, and 84 singletons) with an average age of 60.9 ± 8.3 years (range: 41–78), 25 (7%), 135 (38%), and 198 (55%) of whom were current, former, and never smokers at the time of immunophenotyping, respectively (Figure 1, left panel; Supplementary Table 2). We observed a significant difference in alcohol consumption (ANOVA *p* = 3.9 × 10^−4^) but not in age, body mass index, and socioeconomic status distributions between current, former, and never smokers (ANOVA *p* > 0.05, Supplementary Table 2). In line with studies showing heritability of smoking habits (23–25), monozygotic twin pairs were more likely than dizygotic one to share the same smoking status (72 vs. 55%, Supplementary Table 3).

**Figure 2:**
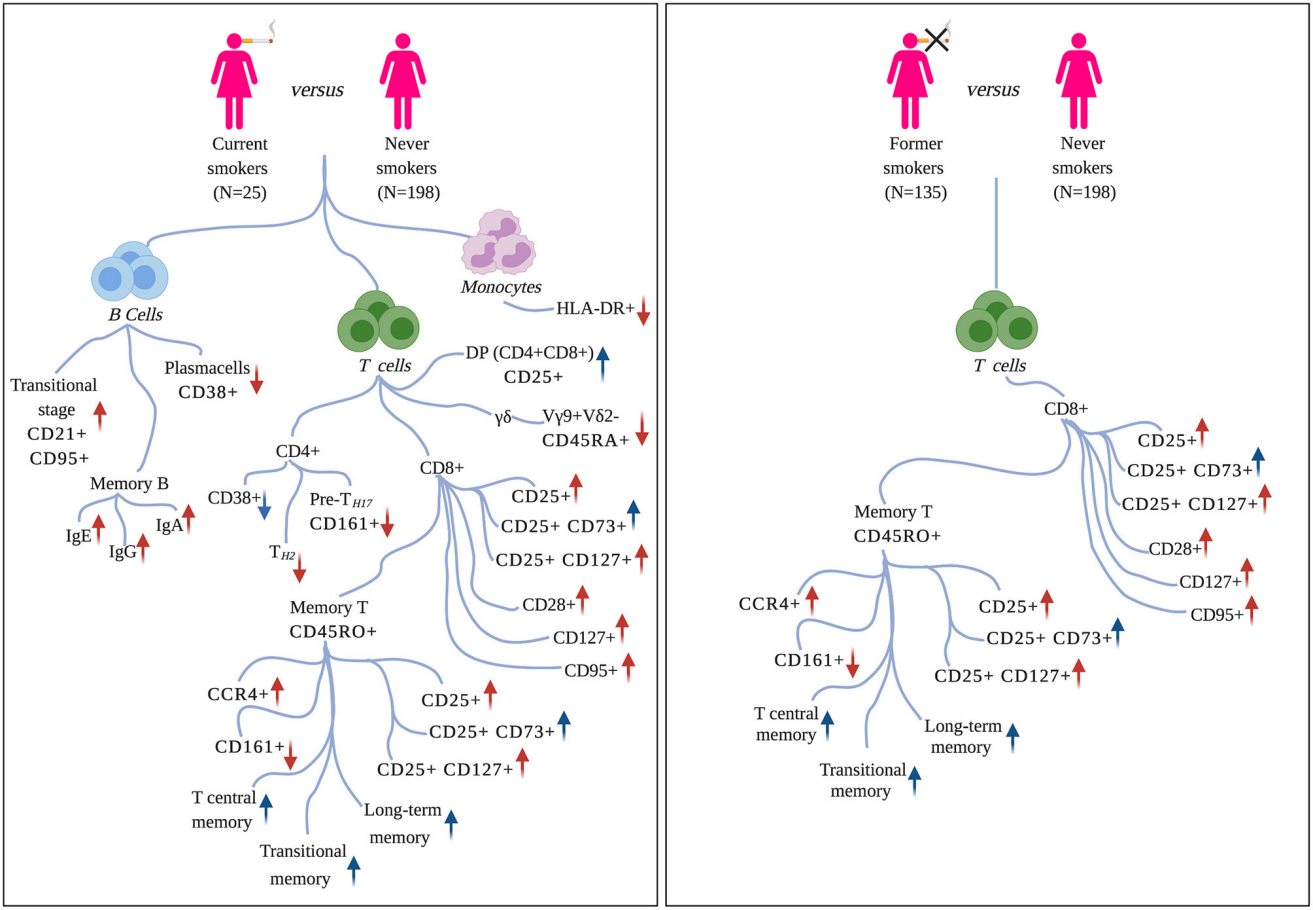
Effects of tobacco smoking on circulating immune traits. **Left panel**: immune traits significantly associated with active smoking (current vs. never smokers). **Right panel**: immune traits that persist altered after smoking cessation (former vs. never smokers). For each molecular marker or cell-subset, arrows summarize the direction of association (positive/negative) and the pro-inflammatory (in red) or immunosuppressive (in blue) function. Markers are defined according to the nomenclature reported in Roederer et al. (18).

Associated immune traits belonging to the same subset were highly correlated to one another (Supplementary Figures 1–3), and this correlation was particularly strong among immune traits presenting similar patterns of molecular markers. This can be explained by the fact that these traits are hierarchically and functionally correlated, as they originated from a limited number of common progenitors. Therefore, to facilitate the description of the obtained results, we present them as groups of highly correlated immune traits characterized by at least a common molecular marker. Mean Pearson's correlation coefficients are reported in the text for each group of CSFs. The cell lineages, subsets, and phenotypic markers used here are defined according to the nomenclature reported in Roederer et al. (18). Our findings are summarized in Figure 2.

### Frequency of Circulating T Cells, B Cells, and Monocytes Is Influenced by Active Smoking

**Figure 3:**
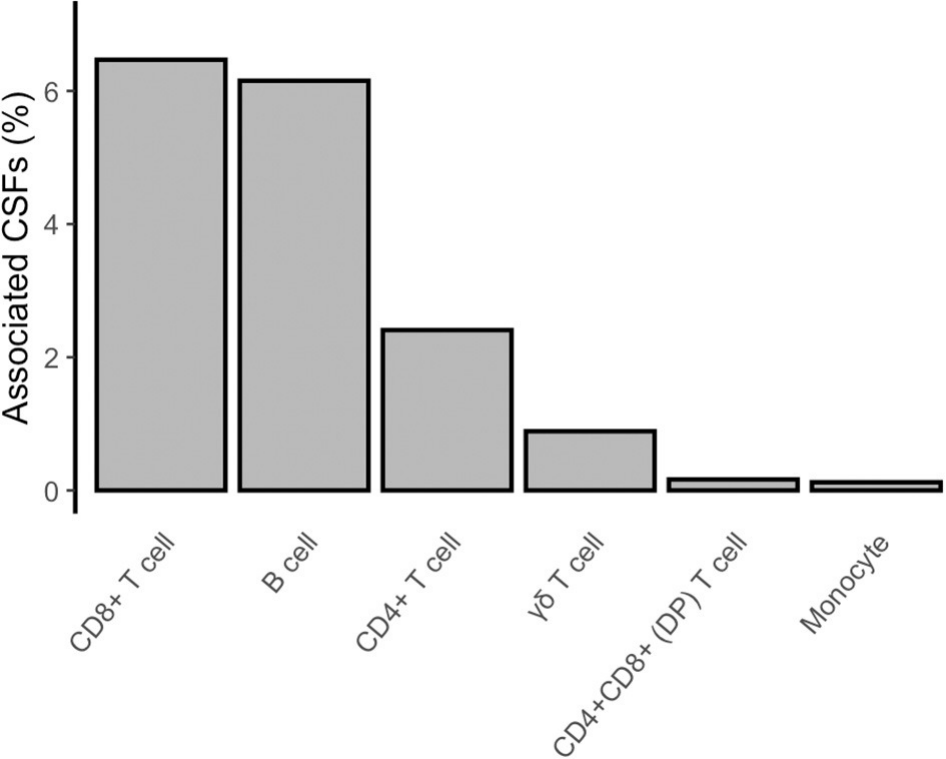
Composition of CSFs associated with active smoking. The bar chart shows, for each group of presented immune trait, the percentage of CSFs associated with active smoking (current vs. never smokers) with respect to the total amount of CSFs analyzed for that group.

In the proposed analysis design, we firstly compared immune trait relative proportions in current (*n* = 25) vs. never (*n* = 198) smokers (section Materials and Methods; Figure 1, right panel). We identified 842 (2%) CSFs associated with active smoking at a Bonferroni-derived threshold of 0.05/2,610 = 1.9 × 10^−5^, and whose association was further confirmed by permutation testing (*P* < 0.05/79 = 6.3 × 10^−4^, section Materials and Methods, Supplementary Data 1, Figure 2, left panel, Figure 3). None of them was significantly associated with alcohol consumption (*P* < 0.05/74 = 6.7 × 10^−4^, section Materials and Methods, Supplementary Data 2), suggesting that the identified associations are attributable to active smoking rather than to the confounding effect of alcohol.

The identified CSFs belonged to the two major lymphocyte populations of T cells (*n* = 710, 84% of the total associated CSFs) and B cells (*n* = 128, 15%), and to the monocyte lineage (*n* = 4, 0.5%). Among the T cells, we found 541 (76% of the associated T cells) CSFs of CD8+, 162 (23%) CSFs of CD4+, 4 (0.6%) CSFs of CD4+CD8+ double-positive (DP), and 3 (0.4%) CSFs of γδ T cells subsets. B cell immune traits included 75 (59% of the associated B cells) CSFs of class-switched memory B cells, 39 (30%) CSFs of plasma cells, 10 (8%) CSFs of transitional, and 4 (3%) CSFs of naïve B cells subsets.

#### CD8+ T Cells

**Figure 4:**
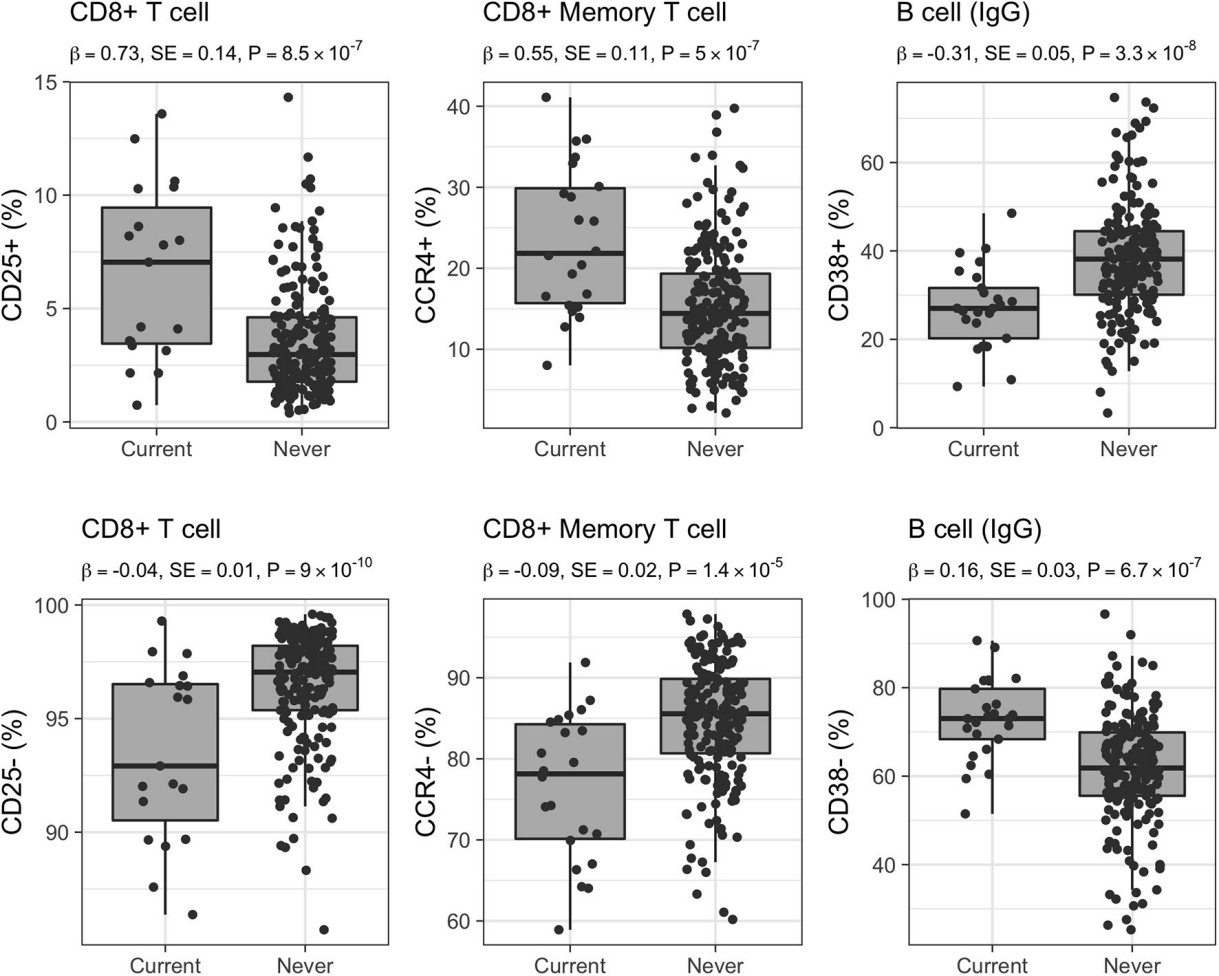
Distribution of selected CSFs in 223 healthy women. Raw relative proportions are plotted, and each boxplot reports effect size (β), standard error (SE), and *p*-value (*P*) of the linear regression analysis (current vs. never smokers). Opposite directions of effects are shown for a selected set of markers: left column CD25 (25+/25–) marker in CD8+ T cells, middle column CCR4 (CCR4+/CCR4–) chemokine receptor marker in CD8+ memory T cells, right column CD38 (38+/38–) marker in B cells isotype IgG.

The largest number of associated CSFs belonged to the CD8+ subset expressing the CD25 activation marker (CD8+CD25+; 280/541, 52% of associated CSFs of CD8+ T cells). More in detail, 65 CSFs expressed exclusively the CD25 activation marker with a pro-inflammatory effect (mean Pearson's |ρ| = 0.90), 52 CSFs expressed CD25 in combination with the CD73 marker with regulatory phenotype and immunosuppressive capability (CD8+CD25+CD73+; mean Pearson's |ρ| = 0.94), and 67 CSFs expressed CD25 and the CD127 marker of proliferation activity and with a pro-inflammatory effect (CD8+CD25+CD127+; mean Pearson's |ρ| = 0.90). The relative proportions of these immune traits were positively associated with active smoking (i.e., their relative proportions were increased in current smokers; Figure 4). Conversely, 4 CSFs of CD8+ T cells not expressing the CD25 marker (CD25-; mean Pearson's |ρ| = 0.94) displayed an opposite direction of effects (i.e., their relative proportions were decreased in current smokers; Figure 4). Within the CD8+CD25+ T cell subsets, we also identified 96 CSFs of CD8+ T cells with memory phenotype (CD8+CD45RO+) positively associated with active smoking. Of these, 34 CSFs expressed exclusively the CD25 activation marker (CD8+CD25+CD45RO+; mean Pearson's |ρ| = 0.98), 32 CSFs expressed the CD25 and CD73 markers (CD8+CD25+CD73+CD45RO+; mean Pearson's |ρ| = 0.99), and 30 expressed the CD25 and CD127 markers (CD8+CD25+CD127+CD45RO+; mean Pearson's |ρ| = 0.98).

Additionally, 88 CSFs of CD8+ T cells with memory phenotype and expressing the CCR4 chemokine receptor were positively associated with active smoking (CD8+CD45RO+CCR4+; mean Pearson's |ρ| = 0.73), whereas 40 CSFs of CD8+CD45RO+CCR4– were negatively associated (mean Pearson's |ρ| = 0.54; Figure 4). We further identified 12 CSFs of CD8+CD45RO+ T cells expressing the CD161 activation and pro-inflammatory marker (CD8+CD45RO+CD161+; mean Pearson's |ρ| = 0.96) that were decreased in current compared to never smokers, and 15 CSFs of central memory T cells (mean Pearson's |ρ| = 0.78), 10 CSFs of long-term memory T cells (mean Pearson's |ρ| = 0.89), and 16 CSFs of transitional memory T cells (mean Pearson's |ρ| = 0.89) that were increased in smokers.

Finally, we observed, in current vs. never smokers, a significant increase in the relative proportions of 17 CSFs of CD8+ T cells expressing the CD28 activation marker (CD28+; mean Pearson's |ρ| = 0.89), 41 expressing the CD127 marker (CD127+; mean Pearson's |ρ| = 0.84), and 18 expressing the CD95 (CD95+, also known as *Fas* or apoptosis antigen 1) marker inducing apoptosis (mean Pearson's |ρ| = 0.85).

#### CD4+ T Cells

We identified 107 CSFs of CD4+ T cells expressing the CD38 activation marker with pro-inflammatory activity (CD38+; mean Pearson's |ρ| = 0.60) negatively associated with active smoking. Conversely, the relative proportions of CD4+CD38– were increased in current smokers (*n* = 8, mean Pearson's |ρ| = 1).

We also identified, as negatively associated with active smoking, a subset of 12 CSFs of T helper-2 (CCR4+, *n* = 12, mean Pearson's |ρ| = 0.91), pre-T helper-17 expressing the CD161 marker (CCR4+ CD161+; *n* = 24, mean Pearson's |ρ| = 0.81), and naïve CD4+ T cells (CD45RA+, *n* = 11, mean Pearson's |ρ| = 0.98).

#### CD4+CD8+ Double Positive T Cells

The relative proportion of 4 CSFs of DP T cells expressing the CD25 activation marker (CD4+CD8+CD25+; mean Pearson's |ρ| = 0.72) was increased in current vs. never smokers.

#### γδ T Cells CD45RA+

The relative proportions of 3 CSFs belonging to the γδ T cells subset (Vg9+Vd2– subset) and expressing the CD45RA naïve marker (CD45RA+; mean Pearson's |ρ| = 1) were decreased in current compared to never smokers.

#### B Cells

CSFs of class-switched memory B cells isotypes IgA, IgG, and IgE (CD38–; *n* = 64, mean Pearson's |ρ| = 0.66; *n* = 10, mean Pearson's |ρ| = 0.65; and *n* = 1, respectively) were positively associated with active smoking. On the contrary, CSFs of B cells expressing the CD38 marker (CD38+; known as plasma cells; *n* = 39, mean Pearson's |ρ| = 0.37; Figure 4) decreased in current smokers compared with never smokers.

We further observed 10 immune traits in the transitional stage of B cells co-expressing the CD21 naïve marker and the CD95 memory B cells activation marker (CD21+CD95+; mean Pearson's |ρ| = 0.85) positively associated, and 4 naïve B cells (CD21+; mean Pearson's |ρ| = 1) negatively associated with active smoking.

#### Monocytes

We identified 4 CSFs belonging to classical monocytes which express CD14 and HLA-DR activation marker and were negatively associated with active smoking (CD14+HLA-DR+; mean Pearson's |ρ| = 0.95).

### Immune Traits Are Either Completely or Partially Restored in Former Smokers

In the second part of our analysis, we investigated whether the 842 CSFs previously identified remained significantly different in former (*n* = 135) vs. never smokers (*n* = 198), that is, whether their relative frequency was restored to non-smoking level in individuals who quitted smoking (section Materials and Methods; Figure 1, right panel). Time since smoking cessation ranged from 1 to 50 years (mean = 22.8, SD = 11.7; Supplementary Figure 4).

**Figure 5:**
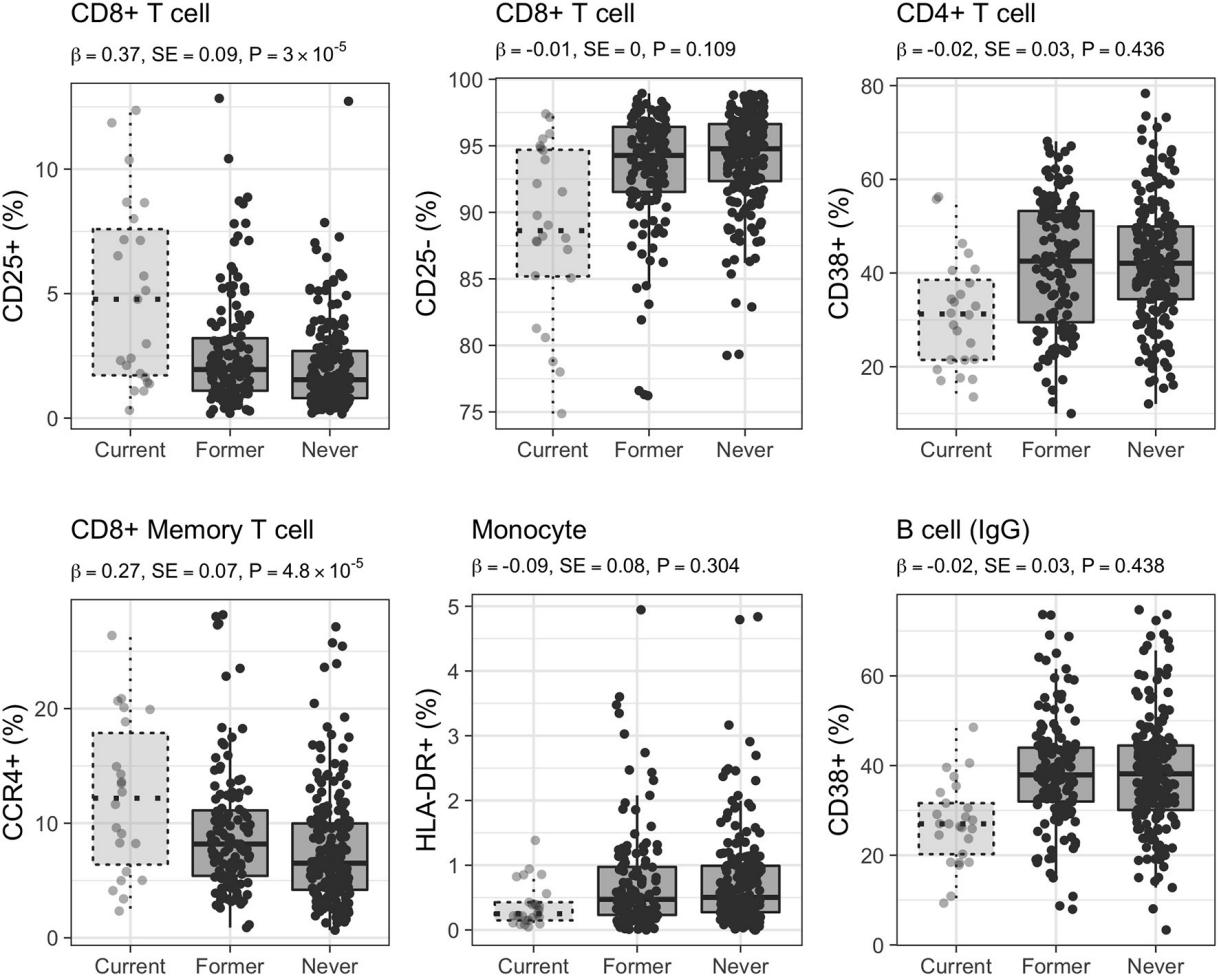
Distribution of selected CSFs in 333 healthy women. Raw relative proportions are plotted, and each boxplot reports effect size (β), standard error (SE), and *p*-value (*P*) of the linear regression analysis (former vs. never smokers). Relative proportions in current smokers are shown for comparison. Left column: CSFs partially restored after smoking cessation, central and right columns: CSFs completely restored after smoking cessation.

Of the 842 CSF associated with smoking, 385 CSFs (46%) were not significantly different between former and never smokers (*p*-value ≥ 0.05), including the majority of CSFs of B cells (120/128, 94%) and a small proportion of CSFs of CD8+ (91/541, 17 %) T cells, and all CSFs of CD4+, CD4+CD8+ DP, and γδ T cells as well as monocyte subsets, suggesting complete restoration after smoking cessation (Figure 5, Supplementary Data 3).

Conversely, at a Bonferroni-derived threshold of 0.05/74 = 6.8 × 10^−4^ (section Materials and Methods), 254 (30%) CSFs, belonging to the CD8+ T cell (254/541, 47%) subsets (further discussed below) continued to be significantly different in former compared to never smokers (Supplementary Data 3, Figure 2, right panel, Figure 5). Interestingly, when the association testing was performed using the three smoking categories (section Materials and Methods, Figure 1, right panel), these 254 CSFs showed significant decreasing effect size from current, to former, to never smokers, thus suggesting that these CSFs were not still completely altered but, in fact, partially restored (Figure 5, Supplementary Data 3).

#### CD8+ T Cells

We observed that 74% (48/65) of the CSFs of CD8+CD25+ T cells remained significantly altered after smoking cessation (Figure 5), as were 55% (37/67) of CSFs of CD8+CD25+CD127+ T cells, 88% (15/17) of the CSFs CD8+CD28+ T cells, 38% (7/18) CSFs of CD8+CD95+ T cells, and 68% (28/41) CSFs of CD8+CD127+ T cells. Conversely, the relative proportion of 17% (9/52) of the CSFs of CD8+CD25+CD73+ T cells, and 50% (2/4) CSFs of CD8+CD25– T cells were completely restored to never smoker levels.

Within the CSFs of CD8+ T cells with memory phenotype, we observed that the relative proportions of all the CD8+CD25+CD45RO+ and CD8+CD25+CD127+CD45RO+ CSFs, and of one CD8+CD25+CD73+CD45RO+ CSF were significantly different in former vs. never smokers, as were 13% (2/15) CSFs of central memory T cells, and all the CSFs of long-term memory T cells and of transitional memory T cells.

CSFs of CD8+CCR4+ and CD8+CCR4– memory T cells showed a mixed behavior: 30% (26/88) of the CSFs expressing the CCR4 chemokine receptor marker (Figure 5) were still altered after smoking cessation, while 36% (32/88) and 90% (36/40) of the CSFs of CD8+CCR4+ and CD8+CCR4– memory T cells were completely restored – as were all the CSFs of CD8+CD161+ memory T cell.

## Discussion

Studies on the effects of tobacco smoking on the immune system have shown conflicting results, most likely because of the variability in smoking exposure (i.e., smoking dose and/or whether cigarettes, pipe, or cigars were smoked) as well as the intrinsic differences in study populations (e.g., in age, sex, or ethnicity), and their sample size (9, 26). Moreover, the majority of these studies focused only on primary leukocyte subpopulations (i.e., CD3, CD4+, CD8+ T cell, B cell, natural killer cells, monocytes, and granulocytes) which are more abundant in blood, and, consequently, easier to measure (5, 12).

In this study, we explored the association between self-reported smoking status and 35,651 immune traits measured by flow cytometry in the peripheral blood of 358 healthy women of European ancestry. To the best of our knowledge, this is the first finely detailed study aimed at elucidating the relationship between smoking and both the innate and adaptive circulating immune cells.

### T Cells

Our study confirmed that active smoking predominantly changes the relative proportions of circulating leukocytes of the T cell subsets. For instance, the increase of CD8+CD25+ T cells reported in our study has been previously observed in the peripheral blood of healthy smokers (27), in the airway epithelium of smokers with chronic bronchitis (28), and in the blood of smokers with lung cancer (29). The identified CD8+CD25+ activated T cells showed a predominance of the proliferating phenotype (CD127+) but also the presence of the regulatory phenotype (CD73+). Studies suggested that CD8+CD25+CD73+ T cell may have an elevate immunosuppressive capacity, inhibiting the proliferation of effector T cells (30, 31), thus explaining the observed pro-inflammatory and immunosuppressive effect of tobacco smoking (5, 6).

Here, we also suggest an association between a small number of CSFs of CD8+CD4+CD25+ DP and of CD8+CD25+ memory T cells and active smoking. While DP T cells are present only in small number in the peripheral blood of healthy individuals, their frequency has been previously associated with several inflammatory and autoimmune diseases (32). In the healthy human thymus, activated CD25+ DP T cells show suppressive functions similar to regulatory T cells (33). The high proportion of CD8+CD25+ memory T cells in smokers is consistent with their increased proliferation following antigen recognition due to a systemic inflammation associated with smoking (34, 35). Taken together, these results suggest that smoking may increase the activation of CD8+ T cells stimulating their proliferation, with concomitant activation of CD8+ T regulatory and DP T cell with immunosuppressive properties. Consistently with the literature, we also observed a positive association between active smoking and CD8+ T cells expressing the CD28 co-stimulatory molecules that play an important role in the activation of the T-cell-mediated immune response during the inflammatory processes (36, 37). Active smoking was also positively associated with CD8+ T cells expressing the CD95 apoptotic marker, previously reported associated with both reduced lung functions and hypoxaemia (38).

We also identified a novel positive association between active smoking and the relative proportion of CD8+ memory T cells expressing the CCR4 chemokine receptor. While the role of the CCR4 receptor in CD8+ memory T cells remains unexplored, studies in animal models showed that cigarette smoking induces the production of CCR4+ ligands in macrophages and dendritic cells, which in turn recruit monocytes in the lung (39) and induce the activation of natural killer cells through the mediation of the CCR4 receptor (40). These findings indicate that the CD8+CCR4+ memory T cells may be involved in smoking response by recruiting CD8+ T cells in inflammatory sites.

We observed an increase of the relative proportion of CSFs of CD8+ memory T cells, in particular central, transitional, and long-term memory subsets, and a decrease of CSFs of T-helper (Th) cells, such as Th2 and pre-Th17 T cell subsets in active smokers. Increase of CD8+ memory T cells and reduction of Th2 in smokers are in line with previous studies indicating a cumulative effect of smoking causing an increase of memory T cells, as well as an immunosuppressive effect decreasing Th-2 response (9, 34, 35). The decrease in the relative proportion of pre-Th17 might be explained by their recruitment into the inflamed lung tissue, where they will exert their pro-inflammatory role as mature Th17 T cells (41, 42).

Furthermore, we observed a decrease of CSFs of CD4+ T cells expressing the CD38 activation marker. This marker is preferentially expressed by naïve CD4+ T cells, conferring a reduced capacity to proliferate and to respond to IL-2 cytokine signaling (43). Interleukin IL-2 carries out homing regulation and proliferation of T cells (44). We speculate that smoking-related inflammation increases IL-2 levels in circulation, promoting the activation of CD8+ T cells, and decreasing the number of CD4+CD38+ T cells in current compared to never smokers. This regulation might be due to an impaired response to IL-2, or the suppressor activity performed by CD8+ T cells with regulatory phenotype induced by smoking (31).

The observed decrease in the relative proportions of CSFs of Vγ9Vδ2 T cells in smokers could be explained by their preferential recruitment in the lung in response to chronic smoke exposure, as suggested by a study in mice (45).

Interestingly, when analyzing data from former smokers, we observed that the relative frequency of CSFs of CD8+ T cells subsets only displayed a partial restoration to non-smokers frequency after smoking cessation. In particular, we found that CSFs of activated CD8+CD25+ T cells, CD8+CD25+ memory T cells, CD8+ T cells expressing the CD28 activation marker and/or the CD127 proliferation marker, those involved in apoptotic pathways (CD95+) as well as CSFs of CD8+ memory T cell expressing the CCR4 chemokine receptor, central memory, long term memory, and of transitional memory T cells were still partially altered after smoking cessation. These results suggest that smoking could persistently alter the phenotypes of the circulating CD8 T cells, and this might reflect permanent damage in the lung tissue caused by long-term exposure. A recent study in former smokers highlighted that the decline in lung functions persists for decades after smoking cessation (46). This decline is consistent with continuous pathophysiological abnormalities, including inflammatory, infective, and immunity abnormalities of the lung in both COPD patients and healthy individuals (9, 47, 48). Moreover, two studies in broncho-alveolar lavage fluid of COPD patients who were former smokers showed that smoking cessation did not prevent the rates of apoptotic T cells from increasing (49), and, even after 5 years, the percentage of CD8+CD25+ T cells in former smokers remained increased in comparison to never smokers (50), further suggesting a persistent inflammation of the airways after quitting smoking.

Conversely, the relative frequencies of CSFs of T reg phenotype (CD8+CD25+CD73+) in both CD8+ T cell subsets, of resting CD8+CD25– T cells, and of CD8+CD161+ memory T cells were completely restored after smoking cessation. Notably, CSFs of CD8+ memory T cells expressing or not the CCR4 chemokine receptor showed a mixed behavior after smoking cessation: those CCR4+ were in equal part completely and partially restored, whilst those CCR4– were almost all completely restored, and we might speculate that this may be again caused by permanent smoking-induced lung damage.

A previous study in peripheral blood on 174 healthy individuals showed that smoking cessation completely restored CD8+ and CD3+ T cell, B cell, and monocyte proportions within 1 year, and CD4+ T cells proportion after 2 years (5), as also observed by other studies which reported either a complete restoration of absolute white blood cell count after 1 year from smoking cessation (51) or of lymphocytes and monocytes count within 2–5 years (52). On the one hand, our results, which show the complete restoration of several cell-subsets, such as B cells and monocytes, confirm these observations. On the other hand, they suggest that the dysregulation of the immune system may persist, at different degrees in other cell-subsets, such as in CD8+ and CD8+ memory T cells. This highlights the importance of investigating the effect of smoking also on finely detailed leukocyte subpopulations, as this may help to explain why the risk of smoking-related pathologies remains elevated even after smoking cessation (53).

### B Cells

Along with the described modification in T cell relative proportions, we observed, in current smokers, an increase of the relative proportions of class-switched memory B cells (IgA, IgG, and IgE isotypes), and a decrease of the relative proportions of plasma cells, the latter releasing immunoglobulins in circulation in response to antigens recognition (54). These findings are in agreement with what reported in a previous study which showed an increase of class-switched memory B-cells isotype IgA in peripheral blood of current smokers compared to former and never smokers (55). The authors suggested that this increase may be the result of chronic inflammation due to continued smoking, and might be associated with the formation or release of (neo)antigens, such as smoke particles or damaged lung tissue. They also hypothesized that continued smoking exposure may cause a secondary immune response, increasing the circulating class-switched memory B cells and memory B cells formation in current smokers (55, 56). As already observed (5, 55), the large majority of CSFs of both class-switched and plasma B cells were completely restored to never smoker levels after cessation.

### Monocytes

Finally, we identified a negative association between active smoking status and CSFs belonging to the monocyte subset. Their decreased relative proportions in smokers could be explained by their preferential recruitment in the lung for generating alveolar macrophages (35), and was completely restored after smoking cessation, in agreement with what reported in the literature (5, 52).

### Limitations

We are aware that this study presents some limitations. First, our study sample included only women of European ancestry, while the effect of smoking on the immune system has been shown to differ between males and females (5, 57) and between European and non-European populations (58). Second, information on smoking status was self-reported while information on second-hand smoking was missing, making it impossible to exclude residual confounding or misclassification bias. Third, the limited sample size hindered the investigation of how the time since smoking cessation influenced the trajectories of immune trait restoration.

### Conclusions

In summary, we detail here how tobacco smoking shapes leukocyte cell subset proportions in healthy women. The shift of immune cells composition in peripheral blood caused by active smoking affects mainly cell-subsets expressing pro-inflammatory or immunosuppressive molecular markers, rather than entire cell-subsets, and changes induced by smoking are not completely reverted even years after smoking cessation. Further investigations are required to dissect the role of these immune traits in smoking- and immune-related diseases, such as autoimmune disorders and cancer.

## Data Availability Statement

The original contributions presented in the study are included in the article/Supplementary Material. Raw data on TwinsUK twin participants are available to *bona fide* researchers under managed access due to governance and ethical constraints. Raw data should be requested via our website (http://twinsuk.ac.uk/resources-for-researchers/access-our-data/) and requests are reviewed by the TwinsUK Resource Executive Committee (TREC) regularly.

## Ethics Statement

This study, involving human participants, was reviewed and approved by St. Thomas' Hospital Research Ethics Committee. The participants provided their written informed consent.

## Author Contributions

GP, MF, and AV designed the study. GP and AV curated the smoking data and conducted the statistical analyses. NR curated the disease status of individuals with immunophenotyping. NR, SC, and AV curated the immune traits data. GP, SR, and DB interpreted the results with contribution from MR, MM, and TDS. GP and AV wrote the manuscript with support from AN, FC, and MF. All authors read and approved the final draft of the manuscript.

## Conflict of Interest

TS is a consultant for Zoe Global Ltd. The remaining authors declare that the research was conducted in the absence of any commercial or financial relationships that could be construed as a potential conflict of interest.
