# Supplementary material for: Immune Trait Shifts in Association With Tobacco Smoking: A Study in Healthy Women
Source: Front Immunol. 2021 Mar 9;12:637974. doi: 10.3389/fimmu.2021.637974 (PMC7985448; doi:10.3389/fimmu.2021.637974)
Supplement: Supplementary file 4 [file Data_Sheet_4.PDF]

# *Supplementary Material*

## 1 Supplementary Data

**Supplementary Data 1. Summary statistics for the associations between immune traits and smoking status (current *vs* never smokers).** For each immune trait (N=35,651), the table reports the immune cell population, lineage, and subset it belongs to, the number of individuals used in the association study (N), and the association study results as effect size (Beta), standard error (SE), p-value (P), and, for those passing the Bonferroni-derived threshold of $0.05/2,610=1.9x10^{-5}$, the empirical p-value from permutation testing (eP). The 842 significant associations are also highlighted (column: significant).

**Supplementary Data 2. Summary statistics for the associations between immune traits and alcohol consumption.** For each immune trait associated with smoking status (current vs never smokers, N=842), the table reports the immune cell population, lineage, and subset it belongs to, the number of individuals used in the association study (N), and the association study results as effect size (Beta), standard error (SE), and p-value (P).

**Supplementary Data 3. Summary statistics for the associations between immune traits and smoking status (former *vs* never smokers).** For each immune trait which was associated with smoking status (current *vs* never smokers, N=842), the table reports the immune cell population, lineage, and subset it belongs to, the number of individuals used in the association study (N), and the association study results as effect size (Beta), standard error (SE), and p-value (P). For immune traits significantly different between former and never smokers ($p<0.05/74=6.8x10^{-4}$, N=254) the results of the association study with the three smoking categories (current *vs* former *vs* never smokers) are reported as the number of individuals included in the study (N.all), effect size (Beta.all), standard error (SE.all), and p-value (P.all).

## 2 Supplementary Tables

**Supplementary Table 1. Molecular markers used to identify the studied immune traits.** For each panel, the table reports the molecular markers used to identify lineages and sub-lineages as well as cells' differentiation and activation stages, and/or their functional capabilities. Markers are listed according to the order used in the gating strategy, with details reported in [doi: 10.1016/j.cell.2015.02.046] (panel 1-5) and in [doi:10.1038/ncomms13850] (panel 6).

| | **Panel 1** | **Panel 2** | **Panel 3** | **Panel 4** | **Panel 5** | **Panel 6** | **Panel 7** |
|---|---|---|---|---|---|---|---|
| | **T cell Differentiation** | **T cell Activation** | **T cell Polarization** | **NK Cell Differentiation** | **γδ T, iNKT-Cells end Hematopoietic Precursors** | **B cell Differentiation** | **Myeloid Cells** |
| Lineage | CD3 | CD3 | CD3 | CD3 | CD34 | CD19 | CD14 |
| | CD4 | CD4 | CD4 | CD4 | CD3 | CD20 | CD11c |
| | CD8 | CD8 | CD8 | CD16 | CD4 | | CD123 |
| | | | | CD56 | CD8 | | HLA-DR |
| Sub-lineage | CD27 | CD25 | CD45RA | | CD1d | IgA | CD1c |
| | CD45RA | CD127 | CCR7 | | TCR-Vγ9 | IgD | CD141 |
| | CCR7 | CD39 | PD1 | | TCR-Vδ1 | IgG | |
| | | CD73 | | | TCR-Vδ2 | IgM | |
| Differentiation, activation stages and/or functional capability | CD28 | CD45RA | CCR4 | CCR7 | CD27 | CD5 | CD8 |
| | CD31 | CD38 | CCR6 | CD62L | CD28 | CD10 | CD16 |
| | CD57 | CCR5 | CXCR3 | CD158a | CD45RA | CD21 | CD32 |
| | CD95 | PD1 | CXCR5 | CD158b | CCR7 | CD24 | CD64 |
| | CD127 | HLA-DR | CCR10 | CD314 | CCR5 | CD27 | CD83 |
| | CD244 | | CD161 | CD335 | | CD95 | CD274 |
| | | | | CD337 | | CD38 | |

**Supplementary Table 2. Sample characteristics.** The dataset includes 358 females of European ancestry belonging to the TwinsUK cohort. Continuous values are reported as mean ± standard deviation, and the presence of a significant difference between the three groups was assessed using the ANOVA test. Data on alcohol consumption and IMD were available for 344 (24 current, 126 former, and 194 never smokers) and 320 (20 current, 123 former, and 177 never smokers) individuals included in this study, respectively. Details on twin pairs concordant/discordant for smoking status are reported in Supplementary Table 3. Of the singletons, 11, 34, and 39 were current, former, and never smokers, respectively.

| | **All** | **Current smokers** | **Former smokers** | **Never smokers** | **P** |
|---:|---:|---:|---:|---:|---:|
| N | 358 | 25 | 135 | 198 | - |
| Zygosity (MZ/DZ/SG) | 86/188/84 | - | - | - | - |
| Age (years) | 60.9 ± 8.3 | 59.8 ± 8.7 | 61.7 ± 8.3 | 60.5 ± 8.3 | 0.33 |
| BMI (kg/m$^2$) | 26.1 ± 4.7 | 24.9 ± 3.9 | 26.3 ± 4.5 | 26.1 ± 4.9 | 0.39 |
| Alcohol (g/day) | 8.2 ± 10.6 | 11.4 ± 12.1 | 10.7 ± 13.3 | 6.2 ± 7.5 | $3.9\times10^{-4}$ |
| IMD | 3.7 ± 1.2 | 3.3 ± 1.4 | 3.8 ± 1.2 | 3.8 ± 1.2 | 0.22 |

MZ: monozygotic; DZ: dizygotic; SG: singleton
BMI: body mass index
IMD: index of multiple deprivations

**Supplementary Table 3. Twin pairs concordant/discordant for smoking status.**

| | | MZ twin pairs | DZ twin pairs |
|---|---|---:|---:|
| | Current-Current | 1 | 0 |
| Concordant | Former-Former | 13 | 12 |
| | Never-Never | 17 | 40 |
| | Total | 31 | 52 |
| | Current-Former | 3 | 6 |
| Discordant | Current-Never | 1 | 2 |
| | Former-Never | 8 | 34 |
| | Total | 12 | 42 |

MZ: monozygotic; DZ: dizygotic

## Supplementary Figures

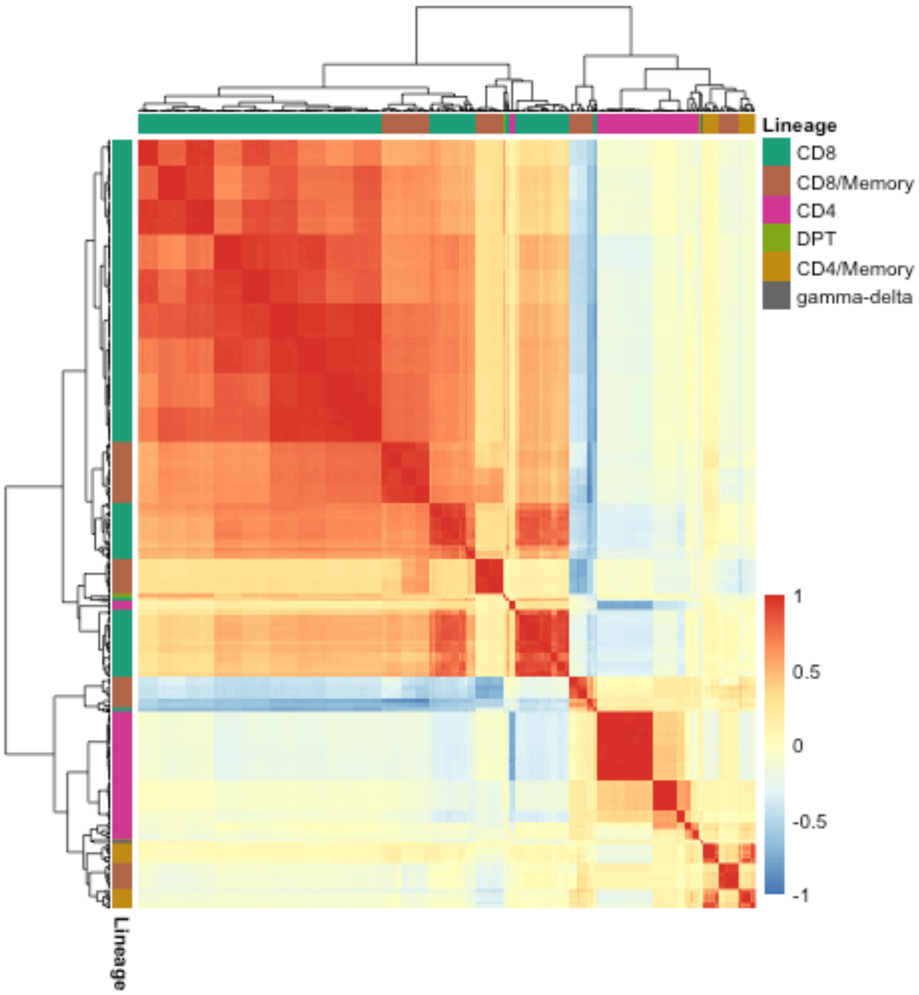


**Supplementary Figure 1. Correlations among T cells associated with smoking status (current vs never smokers).** The heatmap shows Pearson's correlation coefficients evaluated in the dataset of 497 individuals with immunophenotyping. DPT: CD4+CD8+ Double Positive T cells.

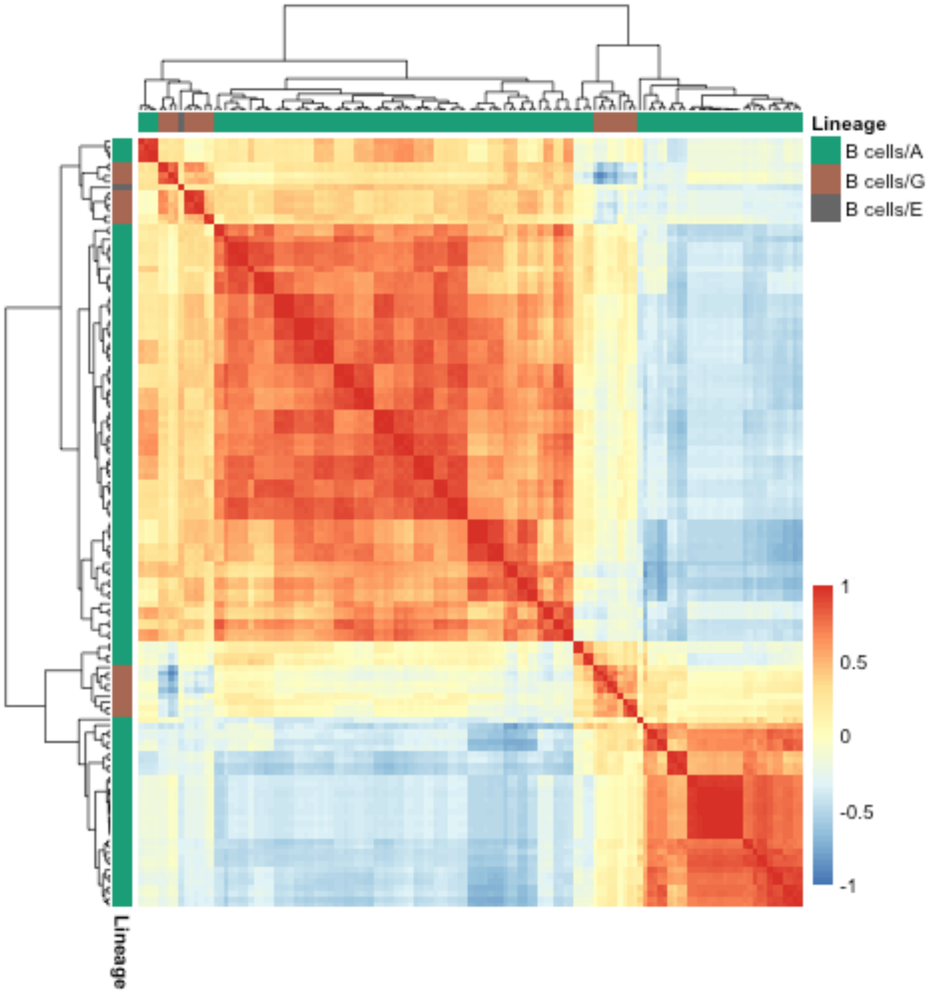


**Supplementary Figure 2. Correlations among B cells associated with smoking status (current vs never smokers).** The heatmap shows Pearson's correlation coefficients evaluated in the dataset of 497 individuals with immunophenotyping. B cell/A: B cell isotype IgA, B cell/G: B cell isotype IgG, B cell/E: B cell isotype IgE.

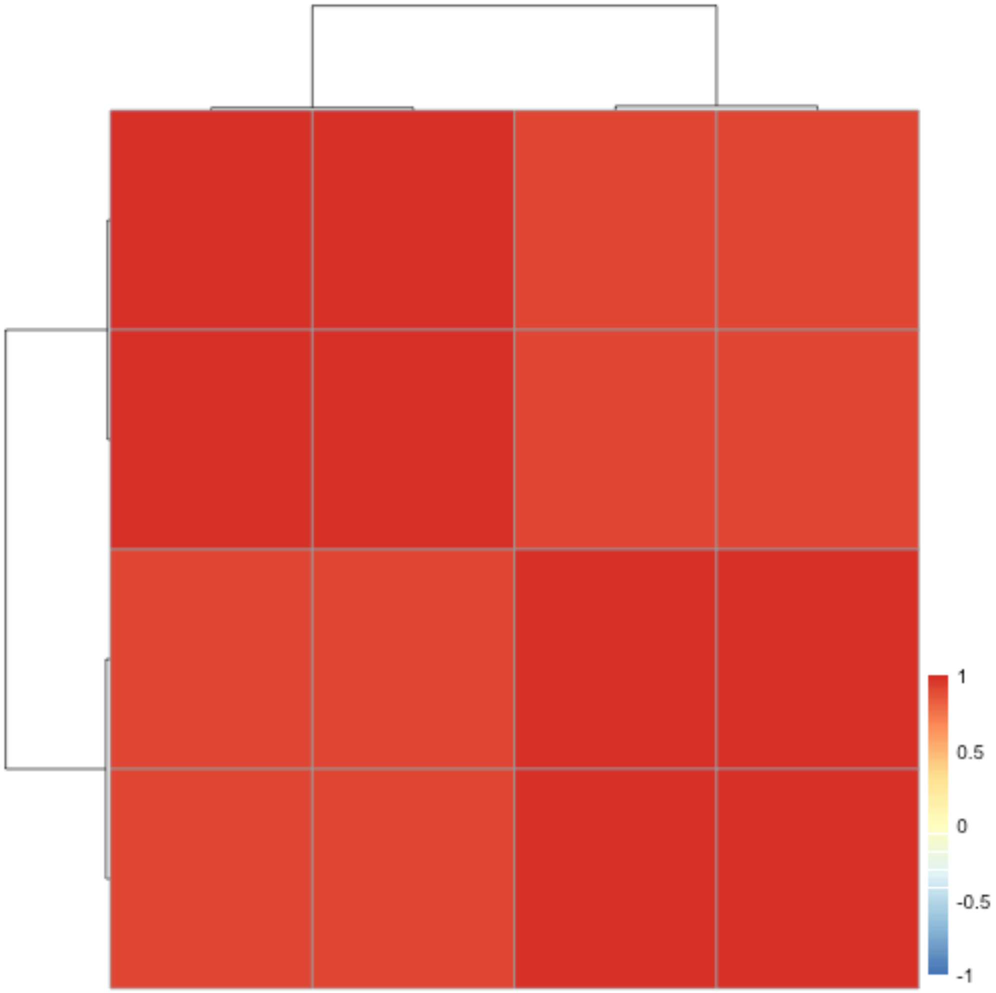


**Supplementary Figure 3. Correlations among monocytes associated with smoking status (current vs never smokers).** The heatmap shows Pearson's correlation coefficients evaluated in the dataset of 497 individuals with immunophenotyping.

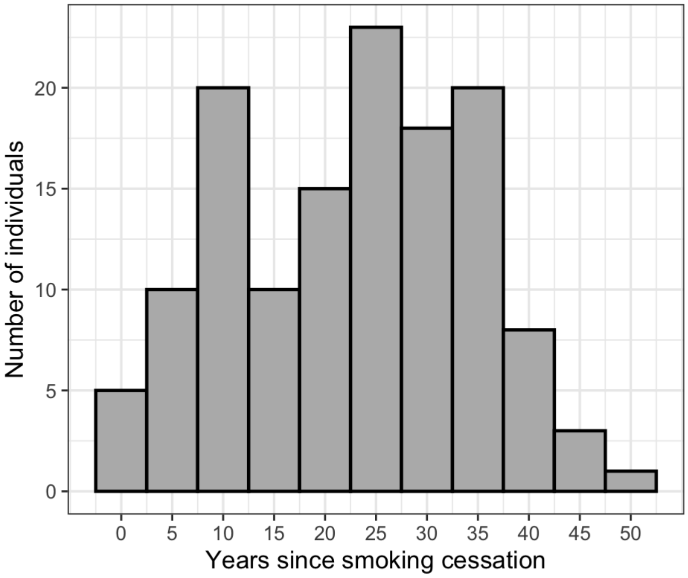


**Supplementary Figure 4. Distribution of years since smoking cessation in former smokers (n=135).**
